# Adaptative Strategy of Immunosuppressive Drugs Dosage Adjustments When Combined With Nirmatrelvir/Ritonavir in Solid Organ Transplant Recipients With COVID-19

**DOI:** 10.3389/ti.2024.12360

**Published:** 2024-03-26

**Authors:** Lidvine Boland, Arnaud Devresse, Caroline Monchaud, Sébastien Briol, Stéphanie Belaiche, Baptiste Giguet, Lionel Couzi, Olivier Thaunat, Laure Esposito, Magdalena Meszaros, Ana Roussoulieres, Vincent Haufroid, Yannick Le Meur, Florian Lemaitre

**Affiliations:** ^1^Department of Clinical Chemistry, Cliniques Universitaires Saint-Luc, Woluwe-Saint-Lambert, Belgium; ^2^Louvain Centre for Toxicology and Applied Pharmacology (LTAP), Institut de Recherche Expérimentale et Clinique, Université Catholique de Louvain, Louvain-la-Neuve, Belgium; ^3^Department of Nephrology, Cliniques Universitaires Saint-Luc, Woluwe-Saint-Lambert, Belgium; ^4^Service de Pharmacologie, Toxicologie et Pharmacovigilance, Centre Hospitalier Universitaire de Limoges, Limoges, France; ^5^ INSERM U1248 Pharmacology and Transplantation, Limoges, France; ^6^ FHU SUPORT, Limoges, France; ^7^ Department of Pharmacy, Lille University Medical Center, Lille, France; ^8^ Department of Hepatograstroenterology, Lille University Medical Center, Lille, France; ^9^ ULR2694-METRICS, Université de Lille, Lille, France; ^10^ Liver Disease Department, Centre Hospitalo-Universitaire Pontchaillou, Rennes, France; ^11^Department of Nephrology, Transplantation, Dialysis and Apheresis, Centre Hospitalier Universitaire de Bordeaux, Bordeaux, France; ^12^Department of Transplantation, Nephrology and Clinical Immunology, Hospices Civils de Lyon, Lyon, France; ^13^Department of Nephrology, Dialysis and Organ Transplantation, Centre Hospitalier Universitaire de Toulouse, Toulouse, France; ^14^Hepatogastroenterology and Liver Transplant Unit, Saint Eloi Hospital, Centre Hospitalier Universitaire de Montpellier, Montpellier, France; ^15^Department of Cardiology, Pulmonary Vascular Diseases and Heart Failure Clinic, Hôpital Erasme, Université Libre de Bruxelles, Brussels, Belgium; ^16^Department of Nephrology, Centre Hospitalier Regional Universitaire (CHU) de Brest, Brest, France; ^17^INSERM UMR1227 Lymphocytes B et Autoimmunité, Université de Bretagne Occidentale, Brest, France; ^18^INSERM UMRS1085, Centre Hospitalier Universitaire (CHU) de Rennes, Rennes, France; ^19^ FHU SUPORT, Rennes, France

**Keywords:** drug-drug interactions, drug monitoring, nirmatrelvir/ritonavir, pharmacokinetic modelling, tacrolimus

## Abstract

Nirmatrelvir/ritonavir is a promising option for preventing severe COVID-19 in solid organ transplant recipients with SARS-CoV-2 infection. However, concerns have arisen regarding potential drug interactions with calcineurin inhibitors (CNI). This two-phase multicentre retrospective study, involving 113 patients on tacrolimus and 13 on cyclosporine A, aimed to assess the feasibility and outcomes of recommendations issued by The French societies of transplantation (SFT) and pharmacology (SFPT) for CNI management in this context. The study first evaluated adherence to recommendations, CNI exposure, and clinical outcomes. Notably, 96.5% of patients on tacrolimus adhered to the recommendations, maintaining stable tacrolimus trough concentrations (C_0_) during nirmatrelvir/ritonavir treatment. After reintroduction, most patients experienced increased C_0_, with 42.9% surpassing 15 ng/mL, including three patients exceeding 40 ng/mL. Similar trends were observed in cyclosporine A patients, with no COVID-19-related hospitalizations. Moreover, data from 22 patients were used to refine the reintroduction strategy. Modelling analyses suggested reintroducing tacrolimus at 50% of the initial dose on day 8, and then at 100% from day 9 as the optimal approach. In conclusion, the current strategy effectively maintains consistent tacrolimus exposure during nirmatrelvir/ritonavir treatment, and a stepwise reintroduction of tacrolimus may be better suited to the low CYP3A recovery.

## Graphical Abstract

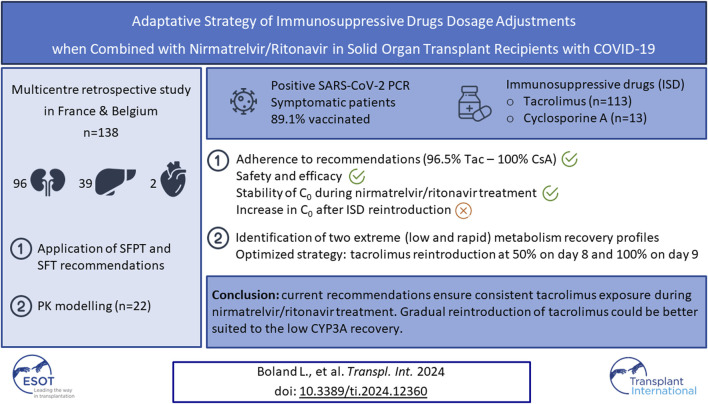

## Introduction

Nirmatrelvir/ritonavir (Paxlovid^®^) is the current first-line treatment to prevent hospitalization and death related to severe acute respiratory syndrome coronavirus 2 (SARS-CoV-2) infection, also known as coronavirus infectious disease 19 (COVID-19) [1]. In the phase III trial EPIC-HR, the drug has been shown to decrease hospitalization and death from severe COVID-19 by 89% for high-risk patients [2]. However, due to the high potency of drug metabolism inhibition of ritonavir, the combination of nirmatrelvir/ritonavir with calcineurin inhibitors (tacrolimus and cyclosporine) and m-TOR inhibitors (everolimus and sirolimus) can lead to their accumulation and subsequent adverse drug reactions, the most worrisome being acute renal failure [3–5]. Despite this potential safety issue, and because the immunosuppressed patients are a high-risk group for severe COVID-19, nirmatrelvir/ritonavir has been prescribed to patients under immunosuppressive treatment with various risk mitigation approaches [6, 7]. In this context, the French societies of Transplantation (Société Francophone de Transplantation—SFT) and Pharmacology (Société Française de Pharmacologie et Thérapeutique—SFPT) have published recommendations to manage immunosuppressants dose adjustment, with the aim of decreasing the risk of accumulation during the nirmatrelvir/ritonavir treatment course in solid organ transplant recipients. In short, these recommendations are: to discontinue tacrolimus 12 h before nirmatrelvir/ritonavir initiation; or to decrease the cyclosporine (CsA) dose to 20% of the initial daily dose and administer it once a day; or to decrease everolimus and sirolimus dose to 12.5% of the initial dose and administer it every other day. For tacrolimus, everolimus, and sirolimus, reintroduction of the dose prior to the course of nirmatrelvir/ritonavir can be considered on day 7, while CsA can be resumed at full dose on day 8 [8]. Specific therapeutic drug monitoring (TDM) of immunosuppressive drugs (ISD) has also been suggested.

The aims of the PAXLOV-IS study were: 1) to evaluate the application of the French recommendations and their impact on exposure to tacrolimus and on clinical outcomes in solid organ transplant patients, and ) to present the results of simulations aimed at proposing an optimized tacrolimus dosage adjustment algorithm when combined with nirmatrelvir/ritonavir.

## Materials and Methods

This two-step retrospective study was conducted in France and Belgium on behalf of the SFT. Between January and August 2022, data on solid organ transplant patients treated with nirmatrelvir/ritonavir from seven French and two Belgian transplantation centers (Bordeaux, Brest, Brussels, Lyon, Montpellier, Rennes, and Toulouse) were collected. Paxlovid^®^ was prescribed to prevent severe complications of SARS-CoV-2 infection in accordance with its summary of product characteristics. The initiation of nirmatrelvir/ritonavir occurred within 5 days after the first symptoms of SARS-CoV-2 infection, for a duration of 5 days, and the dose was adapted to renal function: 150 mg nirmatrelvir + 100 mg ritonavir twice a day if the estimated glomerular filtration rate (eGFR) was below 60 mL/min/1.73 m^2^, or 300 mg nirmatrelvir + 100 mg ritonavir for an eGFR above 60 mL/min/1.73 m^2^.

The following characteristics were collected from medical records and anonymized: sex, weight, age, COVID-19 vaccine status and COVID-19 symptoms, type of transplantation, post-transplantation time, plasma or serum creatinine, glomerular filtration rate estimated using the CKD-EPI formula, liver enzymes, immunosuppressive treatment (type, dose, trough concentrations from baseline to the first measurement after the end of the nirmatrelvir/ritonavir course), and adverse events. The study was authorized by the institutional review board and ethics committee of Limoges Hospital and was registered under #15-2023-03.

### Study Step 1: Application of the SFPT and SFT Recommendations

The first step of this study was to evaluate the application of SFPT and SFT recommendations and their impact on tacrolimus exposure and clinical outcomes, particularly the adverse events potentially related to ISD. The SFPT and SFT recommended interrupting tacrolimus during the 5 days of nirmatrelvir/ritonavir treatment (days 1–5). Reintroduction of tacrolimus was performed at full dose 36 h after the last dose of nirmatrelvir/ritonavir (on the morning of day 7). For CsA, no interruption was recommended, but the dose had to be reduced to one-fifth of the usual dose while on nirmatrelvir/ritonavir and maintained over the 5 days of treatment. The CsA dose was then progressively increased to 50% of the dose administered prior to nirmatrelvir/ritonavir treatment on day 6, 75% on day 7, and full dose on day 8. Other concomitant medications were withdrawn or adapted according to the SFPT recommendations.

### Study Step 2: Pharmacokinetic Modelling

Data were included in the pharmacokinetic (PK) modelling step if at least three trough concentrations (C_0_) were available before, during, and between 8 and 16 days after nirmatrelvir/ritonavir treatment. The pharmacokinetics of tacrolimus were modelled using the MWPharm++ software, as previously described [9]. Individual pharmacokinetic parameters were estimated. Different scenarios were tested to fit the concentration data from the tacrolimus reintroduction period (i.e., day 8–16 period). Tacrolimus areas under the concentration-time curves over 5 days (AUC_0–120h_) were estimated and compared for the 5 days before and the 5 days during nirmatrelvir/ritonavir treatment. The half-life of tacrolimus during nirmatrelvir/ritonavir treatment was also calculated using the following formula:

$$T_{1/2}=\left(\mathrm{Ln}2\;x\;48\right)/\left(\mathrm{Ln}\left(C_{48h}\right)-\mathrm{Ln}\left(C_{96h}\right)\right)$$

where T_1/2_ is tacrolimus half-life, C_48h_ is the estimated concentration of tacrolimus on day 2, and C_96h_ is the estimated concentration on day 4.

The nadir C_0_ before tacrolimus reintroduction and the maximal C_0_ reached during tacrolimus reintroduction were estimated to identify patients with early drug accumulation during tacrolimus reintroduction. Plasma or serum creatinine levels were compared before and at the end of the treatment course. When available, the *CYP3A5* genotype was also gathered and PK parameters were compared between CYP3A5 expressors and non-expressors.

To fit the tacrolimus concentration data measured during tacrolimus reintroduction (from the morning of day 7, 36 h after cessation of nirmatrelvir/ritonavir), different scenarios of metabolism inhibition resolution were applied. This analysis allowed for the selection of the most appropriate strategy for tacrolimus resumption, ensuring sufficient immunosuppressive exposure, while mitigating the risk of drug accumulation. Two extreme scenarios for metabolism recovery were observed in the patients of the study and subsequently tested: 1) a “low metabolism recovery profile” with a progressive metabolism recovery from day 8% to 100% on day 12 and 2) a “rapid metabolism recovery profile,” with a partial (50%) metabolism recovery on day 7 and a complete recovery on day 9.

Then, different strategies of tacrolimus reintroduction were simulated based on a dose regimen of 6 mg once a day: 1) 100% of the dose prior to treatment from day 7, 2) 100% of the dose from day 8, 3) 100% of the dose from day 9, 4) 50% of the dose prior to treatment on day 8, 100% from day 9; 5) 50% of the dose on day 9, then 100% from day 10; and 6) 50% of the dose on days 8 and 9, and then 100% from day 10. An adjudication committee composed of a nephrologist, a clinical pharmacist, and two pharmacologists selected the best scenario to ensure sufficient immunosuppressive exposure while mitigating the risk of drug accumulation.

## Results

### Patient Characteristics

A total of 138 patients were included (63% males), with a median age of 59 years (interquartile range: 48–66). Among them, 96 underwent kidney transplantation (including 3 kidney-pancreas transplants), 39 received liver transplants, and 2 received heart transplants. The majority of patients (121) had undergone transplantation for more than 12 months prior to the study. The median eGFR was 60.5 (IQR: 45.0–77.6) mL/min/1.73 m^2^. Baseline patient characteristics are listed in Table 1.

**TABLE 1:** Baseline characteristics of the studied population.

	Median (IQR)	*N*
Age (years)	59 (48–66)	138
Sex (M/F)	87/50	137
Weight (kg)	66 (57–79.5)	99
Baseline GFR (CKD-EPI mL/min)	60.5 (45–77.6)	138
Tacrolimus daily dose (mg)	3.75 (2.875–6)	112
Cyclosporine daily dose (mg)	120 (100–150)	13

M, male; F, female.

### COVID-19 Infection

A total of 123 patients (89.1%) received two to five doses of mRNA SARS-CoV-2 vaccine prior to COVID-19 infection. The serological response after vaccination was assessed in 82 patients. 20% were non-responders (IgG anti-S < 3 BAU), 23% presented a weak response (IgG anti-S between 3 and 250 BAU), and 57% had a good response (IgG anti-S > 250 BAU). At nirmatrelvir/ritonavir initiation, all patients showed symptoms, including cough (54%), fever (41%), rhinorrhea (38%), sore throat (32%), headache (30%), asthenia (27%), and/or gastrointestinal disorders (7%). All patients had a positive COVID-19 test (PCR). Further genotyping of 35 patients revealed Omicron SARS CoV-2 variants.

### Immunosuppressive Drug Dosing Adjustment

**FIGURE 1:**
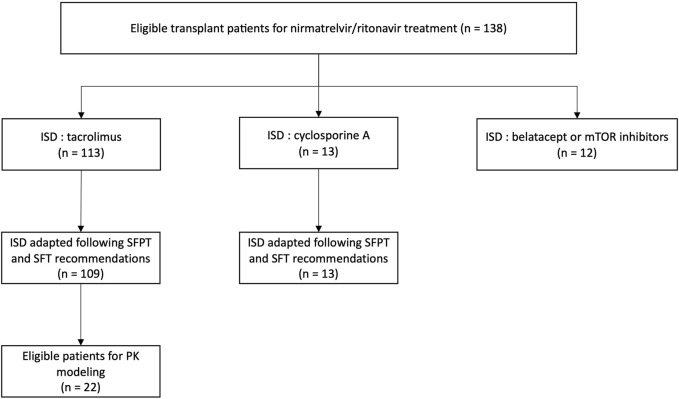
Study flowchart and distribution of immunosuppressive treatment within the population (*n* = 138). ISD, immunosuppressive drug; SFPT, Société Française de Pharmacologie et Thérapeutique; SFT, Société Francophone de Transplantation.

113 patients were on tacrolimus (82%) and 13 on cyclosporine (9%). The remaining patients were on either belatacept or mTor inhibitors and were not included in the analysis (Figure 1).

According to the SFPT and SFT recommendations, all but 4 patients (109/113, 96.5%) discontinued tacrolimus during the 5-day nirmatrelvir/ritonavir treatment: two had a reduced dose of tacrolimus (1.75 mg/d and 0.5 mg/d) and the other two stopped nirmatrelvir/ritonavir before the end due to side effects (digestive intolerance) and resumed tacrolimus on day 4. The SFPT and SFT dose adjustment guidelines were followed for the 13 patients on CsA.

### Trough Concentrations of ISD

**FIGURE 2:**
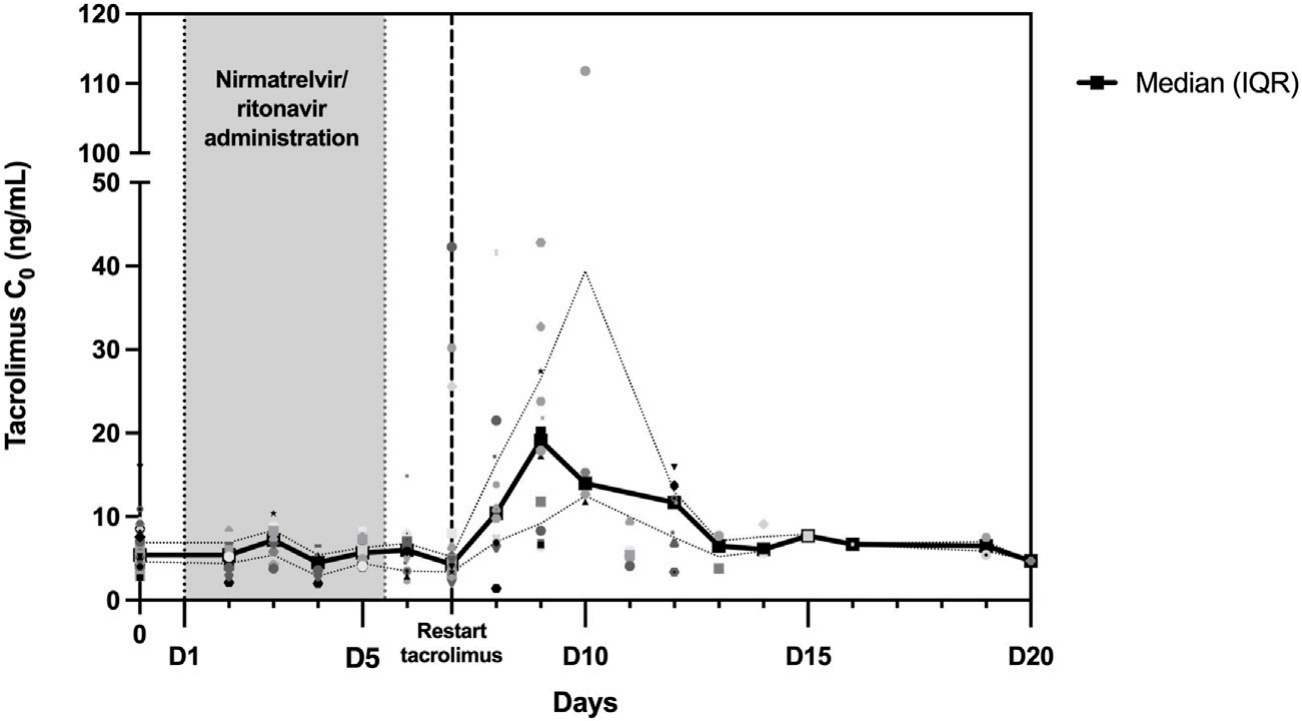
Trough concentrations (C_0_) of tacrolimus during and following nirmatrelvir/ritonavir treatment. Between day 0 and day 20, a total of 192 tacrolimus C_0_ values were collected and are depicted in this figure. The black curve represents the median C_0_, while the dotted curves represent the interquartile range (25th percentile–75th percentile).

Tacrolimus trough concentrations were measured in 103 patients before the introduction of nirmatrelvir/ritonavir. Figure 2 shows the evolution of tacrolimus C_0_ during and after nirmatrelvir/ritonavir administration. TDM was performed in 33 patients after the completion of antiviral treatment (day 6 or 7). For those patients, the median tacrolimus C_0_ remained stable: 5.2 (IQR: 4.3–6.4) ng/mL before nirmatrelvir/ritonavir introduction and 4.4 (IQR: 3.4–5.3) ng/mL before tacrolimus resumption. After tacrolimus reintroduction, C_0_ was monitored in 59 patients: 35 patients between days 8 and 12 and 24 patients after day 12 (between days 13 and 73). In the early reintroduction period (days 8–12), C_0_ increased in most patients with a median tacrolimus C_0_ level of 12.7 (IQR: 6.8–20.9) ng/mL and then normalized. In fact, 15 patients (42.9%) reached concentrations above 15 ng/mL including three (8.6%) above 40 ng/mL. Notably, the highest observed C_0_ exceeded 100 ng/mL; however, this was due to patient error in the tacrolimus dose.

Similar results were obtained for patients on CsA with a median C_0_ of 40 (IQR: 36–70) ng/mL before nirmatrelvir/ritonavir and 111 (IQR: 42–161) ng/mL after full dose resumption.

### Safety and Efficacy

49 adverse events were reported during nirmatrelvir/ritonavir treatment. Dysgeusia was the most frequent symptom (20 patients, 14.5%), followed by diarrhea (17 patients, 12.3%). Adverse events were attributed to tacrolimus toxicity in eight patients (5.8%) (three acute renal failures, two neurologic toxicities, and three gastrointestinal toxicities). One patient for whom the SFPT and SFT recommendations were not followed developed acute renal failure concomitant with a very high tacrolimus concentration. One patient who was treated with CsA experienced acute renal failure. All events were observed in patients with high trough concentrations of ISD and were reversible within a few days after dose reduction.

All patients in this cohort recovered quickly from COVID-19 and none were hospitalized for COVID-19 complications.

### Pharmacokinetic Modelling

Data from 22 patients were included in the modelling step. Table 2 summarizes the treatment and pharmacokinetic parameters of this patient subpopulation. The median tacrolimus C_0_ was 5.2 (IQR: 4.6–6.7) ng/mL before the antiviral course and 4.0 (IQR: 3.4–5.0) ng/mL just before tacrolimus reintroduction (morning of day 7, *n* = 12 patients). The median estimated AUC_0–120h_ before the nirmatrelvir/ritonavir course was 900 (IQR: 684–1,213) ng.h/mL. The median AUC_0–120h_ decreased slightly to 752 (IQR: 622–895) ng.h/mL when tacrolimus was discontinued (i.e., during the antiviral treatment phase). The median decrease in AUC_0–120h_ was 11%. Among the 22 patients, 18 exhibited a decrease in the range of 0%–22%, while the remaining four patients experienced more substantial reductions in exposure, at 47%, 62%, 68%, and 82%, respectively. The median estimated half-life was 212 (IQR: 177–405) hours with some extreme values (range: 87–712 h). The predicted nadir tacrolimus C_0_ in these patients was close to C_0_ prior to the nirmatrelvir/ritonavir course (4.7 vs. 5.2 ng/mL).

**TABLE 2:** Treatment and pharmacokinetic parameters of the 22 patients receiving tacrolimus and included in the modelling part of the study.

	Median	25th percentile	75th percentile
Tacrolimus daily dose (mg)	5	3.375	8.25
Concentration over dose ratio (ng/mL/mg)	0.96	0.75	1.49
AUC0-120 h before N/R (ng.h/mL)	900.1	684.3	1213.3
AUC0-120 h during N/R (ng.h/mL)	752.2	622.2	895.6
Difference in AUCs (before-during) (%)	11%	5%	20%
Half-life during antiviral treatment (h)	212	177	405
Nadir concentration (ng/mL)	4.7	3.8	5.6
Maximum post-treatment concentration (ng/mL)	11.2	8.7	19.2

N/R, nirmatrelvir/ritonavir; AUC0-120 h, area under the concentration time curve between 0 and 120 h.

All patients with available *CYP3A4* genotypes (*n* = 15) were wild-type (CYP3A4*1/*1). Among the 19 patients with an available genotype for *CYP3A5*, 14 were non-expressors (CYP3A5*3/*3) and five were expressors (four CYP3A5*1/*3 and one CYP3A5*1/*1). The half-life did not differ between CYP3A5 expressors (173 h, IQR: 160–294 h) and non-expressors (212 h, IQR: 191–474 h).

PK modelling estimated a median maximal tacrolimus C_0_ of 11.2 (IQR: 8.7–19.2) ng/mL. A maximal C_0_ >10 ng/mL, >15 ng/mL, and >20 ng/mL was estimated in respectively 13 (59%), 8 (36%), and 5 (23%) patients, respectively. However, there was only a slight difference between creatinine measured between days 9 and 16 and creatinine before the antiviral course (median variation: +2.1%, IQR: −3.4–+6.8%), with only three patients reporting an increase in creatinine above +25% of the baseline value (+27%, +31%, and +59%, respectively). None of the 22 patients included in the modelling part of the study was hospitalized for severe COVID-19 or acute renal failure.

**FIGURE 3:**
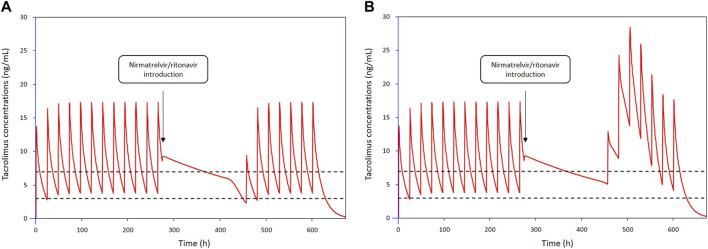
Simulations of tacrolimus blood concentrations for a dosage regimen of 6 mg daily in patients co-treated with nirmatrelvir/ritonavir: **(A)** in a patient with a rapid metabolism recovery and **(B)** in a patient with a low metabolism recovery. Tacrolimus is discontinued from day 1 to day 7 and nirmatrelvir/ritonavir introduced 12 h after the last intake. Tacrolimus is reintroduced on day 8 at 50% of the initial dosing and then 100% from day 9.

Subsequently, the two low and rapid metabolism recovery scenarios and different tacrolimus reintroduction strategies described earlier were tested. The simulated patient receiving a once-daily dose of 6 mg tacrolimus exhibited pre-nirmatrelvir/ritonavir initiation C_0_ of 3.8 ng/mL in the scenario of a rapid metabolism recovery profile, and 5.1 ng/mL in the context of a low metabolism recovery profile. The optimal balance was achieved by reintroducing tacrolimus at 50% of the initial dose on day 8 (60 h after nirmatrelvir/ritonavir last dose) and then 100% from day 9 (84 h after nirmatrelvir/ritonavir last dose). Using this strategy, the estimated nadir of tacrolimus C_0_ after reintroduction was 2.3 ng/mL on the morning of day 8 in the case of a rapid metabolism recovery profile (Figure 3A) and 5.1 ng/mL in the case of a low metabolism recovery profile (Figure 3B). The maximum tacrolimus C_0_ during the reintroduction phase was 3.8 ng/mL on the morning of day 11 in the case of a rapid metabolism recovery profile and 13.8 ng/mL on the morning of day 10 in the case of a low metabolism recovery profile.

## Discussion

We present a collaborative French and Belgian experience, focusing on adherence to the French national recommendations for managing drug-drug interactions between ISD and nirmatrelvir/ritonavir, along with their PK and clinical impact in 138 solid organ transplant patients. Notably, our findings highlight a high adherence rate to the guidelines (96.5% for tacrolimus and 100% for cyclosporine A), revealing sustained tacrolimus exposure but also indicating potential accumulation after early ISD reintroduction.

Nirmatrelvir/ritonavir is a valuable treatment for solid organ transplant recipients with COVID-19 who display a high risk of morbidity and mortality due to SARS-CoV-2 infection. Oral therapy is particularly interesting in outpatient settings. Nevertheless, drug-drug interactions between the antiviral and the immunosuppressive therapy remain a source of concern. The interaction between ritonavir and CYP3A4-dependent drugs can lead to significant increases in drug exposure, up to 50-fold for tacrolimus [10]. Because CNI are highly dependent on CYP3A metabolism, their blood concentration will increase substantially and rapidly when combined with ritonavir. This effect has been previously reported in transplant patients on ritonavir as a single agent or in association [11–13]. High concentrations of tacrolimus can lead to serious side effects such as kidney injury, seizures, posterior reversible encephalopathy, and even death [11]. Several ISD adjustment strategies have recently been reviewed by Tang et al., but no consensus has yet been reached [14]. When CNI are held during nirmatrelvir/ritonavir treatment, studies differ in terms of both timing of ISD suspension and dose resumption. In general, tacrolimus is discontinued from the initiation of nirmatrelvir/ritonavir and resumed at partial or full dose on days 6–13 after treatment completion. Moreover, a close TDM should be considered to guide the resumption of ISD [5, 6, 15–20]. In our study, tacrolimus was discontinued 12 h before nirmatrelvir/ritonavir initiation and restarted at full dose on day 7, while CsA was decreased to 20% of the initial daily dose and resumed at full dose on day 8, according to the SFPT and SFT recommendations [8]. Tacrolimus or CsA trough concentrations were measured during and after nirmatrelvir/ritonavir treatment. This strategy was efficient in the majority of patients. Regarding safety, dysgeusia was the main reported adverse drug reaction, as expected with ritonavir. The second most frequent adverse drug reaction was diarrhea, which was probably of mixed origin (COVID-19 infection, nirmatrelvir/ritonavir, and ISD overexposure). In the whole cohort, four cases of acute renal failure (three in tacrolimus patients and one in CsA patient), two neurologic toxicities, and three gastrointestinal toxicities were reported. These events were consistent with the high exposure reported upon ISD reintroduction. Notably, a deviation from the ISD dosage adjustment was identified in one of these four cases. This is consistent with a recent pharmacovigilance study reporting that 11 out of 14 tacrolimus overexposures were linked to a lack of compliance with the French national guidelines. In two other cases, no information was reported, and only one out of 14 patients seemed to present an overexposure episode while following the guidelines [21]. Fortunately, in our study, all episodes were reversible within a few days with dose adjustments. Furthermore, none of the patients were hospitalized because of severe COVID-19.

**FIGURE 4:**
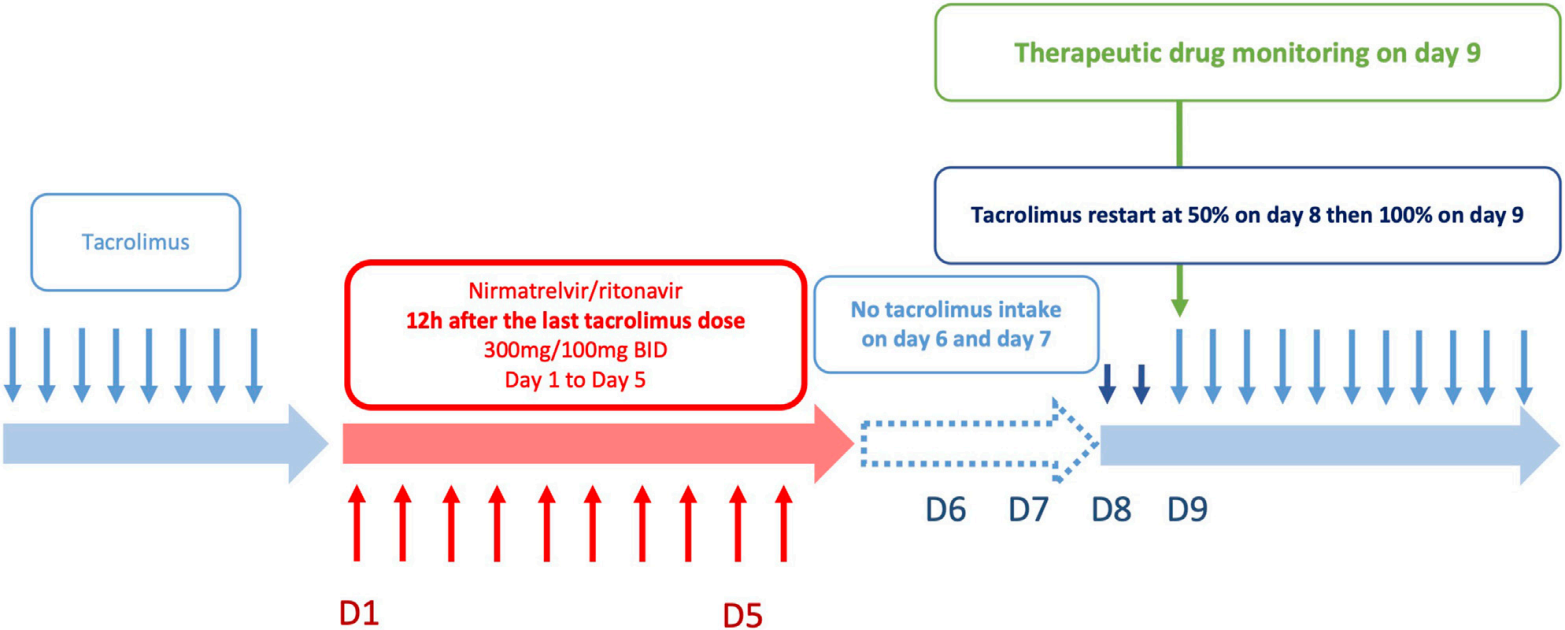
Optimized strategy for tacrolimus reintroduction after nirmatrelvir/ritonavir treatment.

In the second phase of the study, PK modelling was performed in a subset of patients for whom adequate data were available. We showed a sustained tacrolimus drug exposure due to metabolism inhibition during nirmatrelvir/ritonavir treatment, even after tacrolimus discontinuation. Four patients experienced a more pronounced decrease (between 47% and 82%) without any clinical signs of acute graft rejection. However, a considerable number of patients had predicted supratherapeutic levels of tacrolimus after the cessation of nirmatrelvir/ritonavir (C_0_ > 20 ng/mL during days 9–12 in approximately 20% of the patients). Other studies have reported supratherapeutic levels despite tacrolimus interruption during nirmatrelvir/ritonavir treatment [5, 6, 17, 19, 20]. In addition, a few case reports have illustrated the importance of tacrolimus discontinuation to avoid supratherapeutic concentrations and potentially severe adverse reactions [13, 17, 22], sometimes with rifampin [23] or phenytoin [24] treatment for toxicity reversal. Our results suggest a longer inhibition of CYP3A in some patients. Katzenmaier et al. previously reported that it may take at least 3 days after ritonavir discontinuation to restore CYP3A activity [25]. This has led us to re-evaluate the SFPT and SFT recommendations, considering patients’ metabolism recovery. PK modelling of 22 patients allowed us to define two extreme (low and rapid) metabolism recovery profiles. These profiles were used to simulate different strategies for resuming tacrolimus therapy after nirmatrelvir/ritonavir treatment. The best scenario was to stop tacrolimus during nirmatrelvir/ritonavir treatment and restart tacrolimus at 50% of the initial dose from day 8 and then 100% from day 9 (Figure 4). Simulations using extensive data collected from 22 patients showed that this strategy limits the risk of tacrolimus accumulation in patients with a slow recovery metabolism while limiting the risk of low exposure in patients with a rapid metabolism recovery. This one-size-fits-all strategy provides simple and convenient management of this at-risk period following antiviral treatment and is now included in the French national recommendations. Nonetheless, TDM is essential during resumption of immunosuppressive therapy, particularly tacrolimus. This is critical for the early detection of patients who may accumulate immunosuppressants after treatment with nirmatrelvir/ritonavir (days 8–12). Tacrolimus TDM based on trough concentrations measured on days 2 and 3, or even better measurement of its area under the curve, can be proposed to individualize the treatment strategy. Moreover, TDM should be performed early when ISD is restarted to rapidly detect patients with high or low ISD exposure. Volumetric absorptive microsampling (VAMS) could facilitate this process for outpatients infected with COVD-19.

This work has some limitations, including the retrospective design of the study and a limited sample size, especially for the PK modelling phase, where only 22 patients were included. The immunosuppressive treatment in the cohort predominantly consisted in tacrolimus (82%). The observations made on the 13 patients receiving cyclosporine A need confirmation in a larger population, and a similar evaluation should be considered for everolimus and sirolimus. Additionally, it would be interesting to assess the impact of CYP3A genotype on ISD exposure and potential accumulation during nirmatrelvir/ritonavir treatment and after ISD reintroduction.

## Conclusion

This study reports the implementation of the French national recommendations for ISD drug adjustments during nirmatrelvir/ritonavir treatment in 138 solid organ recipients. These data demonstrate that discontinuing tacrolimus 12 h before the introduction of nirmatrelvir/ritonavir enables the maintenance of tacrolimus concentrations within the therapeutic range. It also ensures a tacrolimus exposure during the 5 days of treatment with nirmatrelvir/ritonavir close to the pre-treatment exposure. However, real-life data showed that some patients receiving a combination of tacrolimus-nirmatrelvir/ritonavir experienced tacrolimus accumulation when the treatment was resumed. Simulations performed on patients with repeated TDM showed that a strategy with 50% of the dose initially prescribed from day 8 (60 h after the last nirmatrelvir/ritonavir dose) and then 100% from day 9 (84 h after the last nirmatrelvir/ritonavir dose) should improve drug safety. TDM is an invaluable tool in such combination cases, allowing real-time ISD drug dosage adjustment, and should therefore be used systematically in patients receiving nirmatrelvir/ritonavir.

## Data Availability

The original contributions presented in the study are included in the article/supplementary material, further inquiries can be directed to the corresponding author.

## Abbreviations

AUC_0–120h_, area under the concentration-time curve over 5 days; C_0_, trough concentration; CNI, calcineurin inhibitors; COVID-19, coronavirus infectious disease 19; CsA, cyclosporine A; eGFR, estimated glomerular filtration rate; IQR, interquartile range; ISD, immunosuppressive drugs; PK, pharmacokinetic; SARS-CoV-2, severe acute respiratory syndrome coronavirus 2; SFPT, Société Française de Pharmacologie et Thérapeutique; SFT, Société Francophone de Transplantation; TDM, therapeutic drug monitoring.
